# Osteochondrosis in the feline stifle: a case series and literature review

**DOI:** 10.1177/1098612X241297823

**Published:** 2025-03-20

**Authors:** Sorrel J Langley-Hobbs, Pablo Pérez López, Jess Gower, Karen L Perry

**Affiliations:** 1 Langford Small Animal Hospital, Langford House, University of Bristol, Bristol, UK; 2 Pride Veterinary Centre, Derby, UK; 3 Cave Veterinary Specialists, George’s Farm, West Buckland, Wellington, Somerset, UK; 4 Pat Carrigan Chair in Feline Health, Michigan State University, Veterinary Medical Center, East Lansing, MI, USA

**Keywords:** Osteochondrosis, osteochondritis dissecans, OCD, stifle, patellar luxation, lameness

## Abstract

**Case series summary:**

Information is presented on six new cats with stifle osteochondrosis (OC). In the veterinary literature, there are only four single case reports on cats with this condition. Combining the six new cases with the four previously published cases, we have summarised the current knowledge on stifle OC in the cat. Overall, among the 10 cats, the condition was bilateral in three cats and unilateral in seven. The mean age at presentation was 10.5 months (median 7). Seven cats were male, of which six were castrated, and the three female cats were spayed. Of the cats, five were domestic shorthairs, two were Maine Coons and there was one each of Bengal, Devon Rex and Scottish Fold. The OC lesion affected the lateral aspect of the femoral condyle in seven cats and the medial aspect of the femoral condyle in three cats. All the cats with lesions of the medial aspect of the femoral condyle had unilateral lesions and two of these cats had concurrent medial patellar luxation. All three bilaterally affected cats had lateral femoral condylar lesions. Follow-up of at least 4 weeks was available for 8/10 cats. The lameness resolved or improved in all eight cats: in six cats after surgical debridement of an osteochondritis dissecans (OCD) lesion and in two after conservative management.

**Relevance and novel information:**

Stifle OC or OCD should be a differential diagnosis for immature cats presenting with stifle lameness, stiffness or difficulty jumping and stifle joint effusions. Patellar luxation may be a concurrent diagnosis.

## Plain language summary

Young cats may develop flaps of cartilage in their knees that can cause knee pain, difficulty jumping and stiffness. Changes are seen on radiographs or CT scans. Removal of the flaps should resolve the lameness or rest and anti-inflammatory drugs can also result in an improvement in their gait.

## Introduction

Osteochondrosis (OC) is a focal disturbance of endochondral ossification that can result in a zone of necrotic, weakened cartilage that is susceptible to injury and may lead to flap formation as the fissures extend to the articular surface.^1,2^ Once a cartilaginous flap has formed, the appropriate designation is osteochondritis dissecans (OCD).^1,2^ OCD may lead to synovitis, joint effusion, lameness and degenerative joint disease.^2^ The aetiology of OCD has not been fully identified despite intensive research, but it is considered to be multifactorial with both genetic and environmental factors playing a role.^1,2^ The recent literature strongly supports failure of blood supply to growth cartilage as being the most likely cause.^1^

OCD has been reported in many species including pigs,^3^ dogs,^4^ humans,^5^ horses^5^ and cattle.^6^ In the feline population, OCD has been previously reported in the shoulder,^7–9^ lumbosacral joint^10^ and stifle.^11–14^ In the canine patient, stifle OCD is less common than shoulder or elbow OCD.^15^ In 96% of canine stifles affected by OCD, the lesion is located on the axial aspect of the lateral femoral condyle.^16^ The literature regarding stifle OCD in cats is scarce and limited to single case reports.^11–14^

The aim of this study was to review the literature on stifle OC in cats and compare and contrast this with information on six new cases to increase our knowledge and awareness of this condition.

## Materials and methods

The database at Langford Vets, Bristol Veterinary School, was searched for cases of stifle OC and colleagues at other institutions were contacted to search their databases for cases. To be included, all cases had to have a subchondral femoral defect on radiographs compatible with a diagnosis of OC. Information recorded from the clinical records included signalment, history, clinical signs, radiography reports, concurrent orthopaedic disease, treatment, results of joint fluid analysis and histological analysis of the excised OCD lesion and follow-up. In addition to the new unreported cases, a literature search was performed and information from these data was included in the tabulated and analysed results.

## Results

Six new cats were suspected of or confirmed as having stifle OC and four case reports were identified from a literature search. The six new cases were numbered 1–6 and the four previously published cases were numbered 7–10; these are summarised in Tables 1 and 2. The age range that the cats were first noted to be lame was 5 months–2 years (mean 9.5 months; median 7 months). There were two Maine Coon, one Devon Rex, one Bengal, one Scottish Fold and five domestic shorthair (DSH) cats.

**Table 1:** Signalment, clinical signs and imaging changes of cats with OC of the stifle

Number	Breed, sex, age, weight*	Right or left, lateral or medial aspect of femoral condyle. Bilateral or unilateral	History	Clinical signs	Imaging findings	Concurrent disease	Reference
1	Maine Coon, MN, 7 months, 4.4 kg	R and L lateral. Bilateral	8 months: mild stiffness/lameness L HL started at 7 months after a possible fall off a cat tree. Stretches leg out repeatedly. 13 months: referral to neurology department with persistent L HL lameness 14 months: referral to orthopaedics with history reported as bilateral HL stiffness and unable to jump	8 months: LS discomfort. 13 months: discomfort on L stifle extension. No LS pain. 15 months: pain on stifle extension and bilateral stifle effusions. Mild bilateral HL muscle atrophy. Bilateral MPL grade I–II/IV	Radiography at 7 months: bilateral flattening of the lateral femoral condyles and stifle effusions. Condylar flattening subtle and lesions missed by radiologists. CT at 15 months: L = the lateral femoral condyle is sclerotic with an irregular, 0.4 cm dish-shaped defect in the subchondral bone of its caudal portion. Sitting within this defect are several small mineral fragments. – The trochlear groove is subjectively slightly shallow, though the patella sits centrally within it. – A mild stifle joint effusion is noted. R = the lateral femoral condyle is sclerotic with an irregular, 0.45 cm dish-shaped defect in the subchondral bone of its caudal portion. – The trochlear groove is subjectively slightly shallow, though the patella sits centrally within it. – A very mild/equivocal stifle joint effusion is noted. Conclusions: – Bilateral OC of the lateral femoral condyle, with presence of joint mice on the L side. – Bilateral joint effusion (mild on the L and very mild on the R)	Bilateral grade I–II/IV MPL	
2	DSH, MN, 5 months, 3.2 kg	R medial. Unilateral	R HL lameness with a 1-month duration	Moderate R HL lameness and palpable patella luxation. Thickening of R stifle, positive cranial draw but with abrupt end compatible with normal laxity in young cats. Positive Ortolani R hip	Radiographs at 6 months: large subchondral erosion on the R medial femoral condyle. Patella displaced medially compatible with a grade II or III MPL	Unilateral grade II–III/IV MPL	
3	Scottish Fold, MN, 10 months, NR	R lateral. Unilateral	Marked R HL lameness	Grade III/IV lame R HL. Stifle swollen and painful No instability	Mild flattening of the lateral femoral condyle and joint effusion. Intra-articular mineralisation	None	
4	Maine Coon, MN, 7 months, 2.95 kg (BCS 3/9)	R lateral. Unilateral	Mild R HL lameness	Mild R HL lameness with a tail shift. There was moderate, circumferential swelling of the R stifle. The R stifle was painful upon flexion and extension and crepitus was palpable. There was moderate muscle atrophy of the R HL. Under sedation there was mild instability in cranial draw on the R and L stifles, both with an abrupt endpoint and consistent with the immature age of the patient	Large concavity in subchondral bone margin of lateral femoral condyle with substantial sclerosis affecting most of lateral condyle. Severe stifle effusion. Mineralisation lateral to lateral condyle on oblique Cr-Cd view. Physes open consistent with age	None	
5	DSH, FN, 8 months, 3.5 kg	L lateral. Unilateral	L HL lameness with a duration of 10 days. No known injury and the cat was still playing and running through the home	Mild swelling of L stifle and palpable patella luxation	Large concavity in subchondral bone margin of L lateral femoral condyle with mild associated sclerosis. Moderate stifle effusion. Small area of mineralisation cranially within joint at cranial extent of tibial plateau – 2 mm diameter. Mineralised flap evident distal to lesion on Cr-Cd view. R stifle no abnormalities noted	None	
6	Devon Rex, M, 9 months, NR	R and L lateral. Bilateral	Acute onset L HL lameness with a duration of 4 months. NWB lame initially but then improved. Ten weeks later, it developed R HL lameness that resolved rapidly. Bunny hopping occasionally and lost ability to jump	Mild tarsal hyperextension worse on the L. No obvious lameness at a walk. Pain on extension of the L and R stifles. Mild HL muscle atrophy	Bilateral stifle effusion with mineralised ossicles at the caudal aspect of both stifles. Flattening of the lateral aspects of the femoral condyle	None	
7	DSH, MN, 9 months, NR	R and L lateral. Bilateral	Progressive R HL lameness with a 2-month duration – surgery. It developed L HL lameness 7 months later	R: severe lameness with pain on flexion and extension of the R stifle	Subchondral defect and flattening of the lateral aspects of the femoral condyle with subchondral sclerosis. A moderate amount of soft tissue swelling and faint mineral fragments in the caudal aspect of the stifle joints	None	11
8	DSH, MN, 11 months, 3.9 kg	R lateral. Unilateral	Sudden onset R HL lameness with a duration of 14 days. Mild improvement on rest and NSAIDs. Stiffness after rest and progressive deterioration	2/10 R HL lameness. Periarticular thickening and effusion palpable	Irregular subchondral bone defect and flattening of the lateral aspect of the R femoral condyle. R femoral condyle appeared truncated when compared with the L. Sclerosis present adjacent to the subchondral bone defect. Joint effusion. Small irregular mineral opacity (2 × 4 mm) present in cranial joint space	None	12
9	Bengal, FN, 24 months, NR	L medial. Unilateral	Intermittent, mild L HL lameness, which the owner reported to be associated with an audible click	Luxation of the L patella with flexion of the stifle and concurrent internal rotation of the tibia that spontaneously repositioned with stifle extension. An effusion of the L stifle, and signs of mild resentment to extension of the coxofemoral joints bilaterally were also present. Palpation of the R stifle was unremarkable	Mild bilateral hip dysplasia with early signs of osteoarthritis. Orthogonal radiographs of the L stifle revealed a well-defined, smoothly marginated mineral opacity within the craniomedial aspect of the intercondylar notch, 3 mm × 1 mm in size, distal femoral varus, a medially positioned patella within the femoral trochlear, and new bone formation on the distal pole of the patella. Orthogonal views of the R stifle revealed distal femoral varus and a fine mineralised streak in the craniomedial aspect of the intercondylar notch. The R patella was positioned centrally in the femoral trochlear	Unilateral MPL grade II/IV	13
10	DSH, FN, 7 months, NR	L medial. Unilateral	Two-month history of progressive HL gait abnormality, potentially caused by entanglement in holiday lights	No overt lameness, mild discomfort on manipulation of the L stifle, and grade II/IV R and III/IV L MPL	Moderate (R) and marked (L) effusions; medial displacement of the L patella; and a large, well-defined, subchondral bone defect within the L medial femoral condyle with linear mineralised fragments distal to the defect	Bilateral grade II/IV (R) and III/IV (L) MPL	14

*Age at first clinical signs, or first presentation, whichever was younger, weight at first presentation BCS = body condition score; Cr-Cd = craniocaudal; DSH = domestic shorthair; F = female; HL = hindlimb; L = left; LS = lumbosacral; M = male; MPL = medial patellar luxation; N = neutered; NR = not recorded; NSAID = non-steroidal anti-inflammatory drug; NWB = non-weightbearing; OC = osteochondrosis; OCD = osteochondritis dissecans; R = right

**Table 2:** Treatment and results of investigations and follow-up in cats with OC of the stifle

Number	Treatment and surgical findings	Arthrocentesis	Histological analysis of debrided cartilage flaps	Duration of follow-up and outcome	Reference
1	Bilateral lateral parapatellar stifle arthrotomies and removal of OCD lesions and curettage of subchondral bone. Lateral retinacular imbrication for MPL	Compatible with degenerative arthropathy	Hyaline cartilage with a focal area of abrupt mineralisation and atypical ossification. These findings, in conjunction with the lack of an inflammatory cellular infiltrate or other pathological changes, would be compatible with OC or osteochondritis	Lameness persisted between 8 and 15 months of age despite conservative management with meloxicam and crate rest. 5-week postoperative follow-up: wounds healed well without any complications. ‘He appears to be in much less pain than he was. Demeanour is very much like when he was on pain relief before, suggesting the pain reduced. He does not stretch his back legs out behind him as much as he did albeit this hasn’t stopped and when he does he will generally stretch out his left leg. He will also sit with his knees bent which he never did before. He still has weakness when jumping and climbing the stairs.’ 5-month follow-up: full recovery from HL stiffness and weakness. ‘He has built up his back legs and can jump onto surfaces without any trouble. He seems far happier in himself and loves to play.’	
2	Unilateral lateral parapatellar stifle arthrotomy and removal of OCD lesion (one large piece and two smaller pieces) from lateral aspect of medial condyle adjacent to origin of caudal cruciate ligament. Minimal curettage of subchondral bone. Cranial cruciate ligament intact. Block recession and lateral imbrication for MPL	0.4 ml of pinkish tinged fluid R stifle. 1 drop clear fluid L stifle. Aerobic and anaerobic culture negative	0.5 × 0.3 cm lesion. Central areas of osseous tissue but the majority is of peripheral chondroid elements of less mature appearance and with marginal attachments of more fibrocartilaginous and fibrous tissue. The central osseous area is subject to section fragmentation artefact. Cartilage areas contain chondroblasts as large cells in copious matrix. Peripheral connective tissues are compressed. An inflammatory process is not present	2-month follow-up: cat back to normal indoor/outdoor activity. Radiographs showed healed osteotomy and patella centrally located	
3	Unilateral lateral parapatellar stifle arthrotomy with OCD lesion removal and debridement of subchondral sclerotic bone	NP	NP	2-week postoperative follow-up: incision healing well without signs of infection. Then LTFU	
4	Rest and NSAIDs	NP	NP	Follow-up at 6 years 7 months: owner states that the cat can go up and down stairs without any issues and jumps up on the counter to reach for his treats. When he runs it does look like his leg is a little stiff, but it does not appear to bother him	
5	Rest and NSAIDs. Followed by a gradual return to normal activity	NP	NP	12-month follow-up: lameness reported as resolved. Repeat radiograph at 12-month follow-up (Cr-Cd view), lesion not clearly visible. No radiographic evidence of OA but no lateral view so unable to determine if effusion. At 3-year follow-up, the cat was reported as doing well: running and playing well with no difficulty. Occasionally a little stiff on the L HL but still able to jump onto surfaces to eat treats. No lameness appreciated on gait assessment. Grade I MPL bilateral. L stifle slightly thickened. Full range of motion of both stifles with no associated pain response and no palpable instability. The remainder of the orthopaedic examination was within normal limits. Bilateral stifle radiographs at 3-year follow-up: R = Small area of mineralisation within the cranial joint compartment – likely incidental and the sesamoid bone within the medial meniscus is not associated with any effusion or other radiographic changes. L = Large OCD fragment noted in the caudolateral aspect of the joint. Severe flattening of the lateral femoral condyle – more prominent on the mediolateral view than the craniocaudal. Moderate periarticular osteophytosis. Mineralisation within the cranial aspect of the joint, larger and more irregular than the incidental findings noted on the R	
6	LTFU	LTFU	LTFU	LTFU	
7	Bilateral staged arthrotomies and OCD lesion removal and curettage. RJ 2 weeks and cage rest postoperatively	NP	R stifle: hyaline articular cartilage containing irregular zone of mineralised cartilage in the deep aspect parallel to the articular surface. Immediately subjacent to the zone of mineralised cartilage was a zone of chondrocyte clones. Subjacent to the chondrocytes, and forming the deep margin of the tissue, was a thin zone of necrotic cartilage. The histopathological appearance of this specimen was essentially identical to that of cartilage flaps removed from dogs with OCD.^1,2^ L stifle: nodule of bone surrounded by fibrillated fibrocartilage or hyaline cartilage, depending on the location within the section	14 months – sound	11
8	Arthroscopic assisted debridement of fragments	Consistent with degenerative arthropathy	Central core of hyaline cartilage with variable extensive mucosis with mineralisation. Surrounding the core was a variable thick cuff of proliferative cartilage, well-differentiated chondrocytes being arranged as variably sized chondrocytes with territorial matrix. Surrounding the middle zone of proliferative cartilage was a narrow cuff composed of bland appearing spindled mesenchymal cells with a fibrous stroma. These findings were consistent with a loose body	10-month follow-up: complete resolution of lameness. Able to jump. Owner reported the cat to be reluctant to flex stifle when sitting	12
9	Arthroscopy of the L stifle using a 1.9 mm arthroscope via a standard craniolateral scope portal and craniomedial instrument portal. Medial patella luxation evident with flexion of the stifle. Full thickness 4/5-modified Outerbridge erosion approximately 3 mm in width was present on the axial border of the medial femoral condylar notch. During the examination, a well-defined osseous fragment floated free from the instrument portal	NP	The loose fragment was submitted for histopathology, which found the section to be composed of dense aggregates of fibrocartilage, within which there was extensive endochondral ossification present as well as poorly organised chondrocyte columns towards the periphery of the tissue, many of which were organised in dense, irregular clusters towards the articular surface. There was irregular mineralisation of the chondroid matrix supporting these clusters of chondrocyte columns; this was consistent with an osteochondral fragment associated with OCD of the medial femoral condyle but could also have been consistent with intra-articular ossification of soft tissues, such as the fat pad	Lameness persisted and 2 weeks later the cat was anaesthetised and a tibial tuberosity transposition and lateral fascial imbrication were performed to treat the MPL. Six weeks after MPL surgery, the lameness had resolved with normal patella function palpable in the L stifle	13
10	Bilateral lateral parapatellar stifle arthrotomies were performed. On the left, there was a large, loosely adhered portion of cartilage on the medial femoral condyle. The cartilage flap was removed and the underlying subchondral bone was debrided. For the treatment of MPL, bilateral antirotational fabellotibial sutures were placed using 0 polypropylene, and on the left, a lateral fabellopatellar suture was placed using 1 polydioxanone	L = increased volume of joint fluid containing an increased number of mononuclear cells consistent with a non-inflammatory arthropathy	Fragments of cartilage and mineralised cartilage consistent with an OCD lesion	4-week follow-up progressive improvement in limb use and no signs of discomfort. No discomfort on stifle manipulation but patellae would luxate on stifle flexion	14

Cr-Cd = craniocaudal; FU = follow-up; HL = hindlimb; L = left; LTFU = lost to follow-up; MPL = medial patellar luxation; NP = not performed; NSAID = non-steroidal anti-inflammatory drug; OA = osteoarthritis; OC = osteochondrosis; OCD = osteochondritis dissecans; R = right; RJ = Robert Jones bandage

Three cats were affected bilaterally and seven unilaterally, with a total of 13 affected stifles in the 10 cats. The presenting problem was one of unilateral or bilateral lameness with an inability or reduced ability to jump mentioned for two cats. The duration of lameness before presentation, diagnosis or treatment of OC was in the range of 2 weeks–6 months (median 2 months). Onset varied from acute, associated with a possible low-grade trauma such as a fall, to intermittent or insidious progressive lameness. The common changes on clinical examination included stifle swelling and pain on manipulation (extension and flexion).

Radiographic changes included a subchondral defect on, or flattening of, the lateral or medial aspect of the femoral condyle in all cases, stifle effusions and intra-articular mineralisation or mineralised bodies. In two of the unilaterally lame cats there were radiographic changes in the contralateral stifles (joint effusions and mineralisation). The lateral aspect of the femoral condyle was affected in 10 stifles and the medial aspect in three. The lateral aspect of the femoral condyle was affected in the three bilateral cases.

Four cats had concurrent medial patellar luxation, ranging from grade I to grade III;^17^ in two cats this was bilateral. In the two cats with unilateral patellar luxation, the osteochondral lesion was on the medial aspect of the femoral condyle.

Two cats were treated conservatively and seven cats had surgery (Table 2). Surgery consisted of OCD flap removal and some degree of curettage of the underlying subchondral bone. Two of the surgically treated cases were treated arthroscopically and five via arthrotomy. One of the arthroscopically treated cases had an arthrotomy performed at a later date to treat persistent lameness presumed to be due to the previously diagnosed medial patellar luxation.

Arthrocentesis was performed in four cats (Table 2) and the results were consistent with a degenerative or non-inflammatory arthropathy, with an increase in mononuclear cells. The OCD flap was submitted for histopathological analysis in six cats. The results were consistent with OC (Table 2).

Follow-up of at least 4 weeks was available for eight cats. The two cats treated conservatively had a mean follow-up of 4 years 8 months and both cats were able to run and jump but were occasionally reported to be stiff on the affected leg. Seven of the cats treated surgically had a mean follow-up of 5 months (range 4 weeks to 14 months). All of the cats treated surgically were reported to have no lameness and to have returned to normal activity. One cat did not flex its stifle when sitting and in one cat the pre-existing patellar luxation persisted postoperatively.

**Figure 1:**
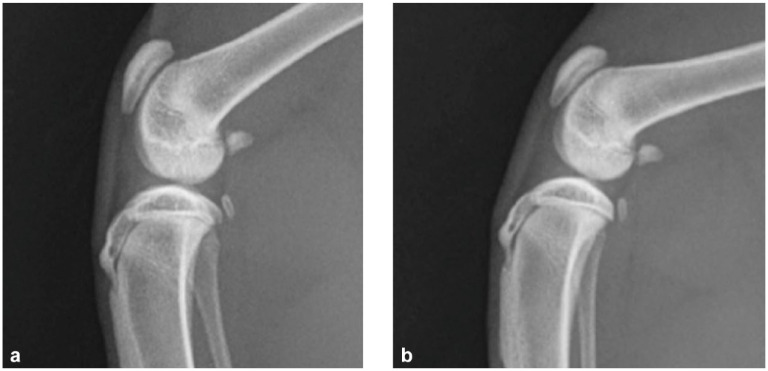
Mediolateral views of the (a) right and (b) left stifles of case 1, a male castrated Maine Coon cat at 8 months of age. Bilateral joint effusions and subtle flattening of the lateral femoral condyles can be seen

One cat that had bilateral OCD, radiography and a CT scan, surgery and follow-up is described in detail. A male castrated Maine Coon cat weighing 4.4 kg had a sudden onset of lameness at the age of 7 months, possibly associated with a fall from a cat tree. An initial improvement had been seen on rest and meloxicam (Metacam; Boehringer Ingelheim) but clinical signs persisted. Radiographs taken at the referring veterinary surgery when the cat was 8 months of age showed bilateral stifle effusion. There was subtle flattening of the lateral femoral condyles bilaterally (Figure 1). On clinical examination, the problem was difficult to localise but it was felt that there was some resentment of palpation of the lumbosacral spine.

A persistent failure to improve resulted in referral to the neurology department at Langford Vets. At this visit, no lumbosacral pain was detected and the cat was discharged home with a further period of rest and non-steroidal anti-inflammatory drugs (NSAIDs). The cat was re-examined 6 weeks later in the feline orthopaedic clinic at 15 months of age. The cat was not noticeably lame but there was consistent discomfort on stifle extension bilaterally, stifle swelling and low-grade patellar laxity (right grade I/IV, left grade I–II/IV).

**Figure 2:**
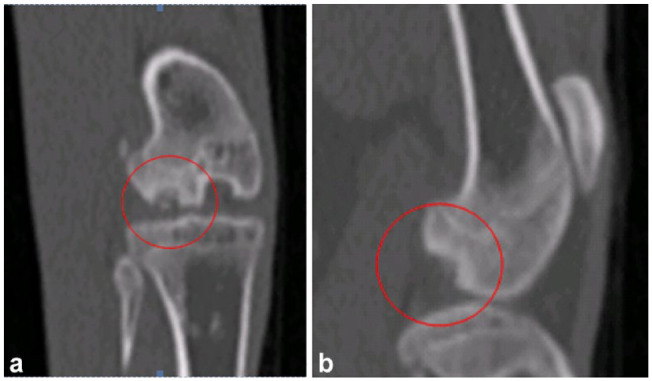
CT scans of case 1, a male castrated Maine Coon cat at 15 months of age: (a) the left lateral femoral condyle is sclerotic with an irregular, 0.4 cm dish-shaped defect in the subchondral bone of its caudal portion. Sitting within this defect are several small mineral fragments. (b) The right lateral femoral condyle is sclerotic with an irregular, 0.45 cm dish-shaped defect in the subchondral bone of its caudal portion

After routine sedation, CT scans were obtained and arthrocentesis performed of both stifles. The CT scan showed subchondral defects on the lateral aspect of the condyle of both femora and there were bilateral mild joint effusions. The changes were compatible with OC (Figure 2). Samples of joint fluid were obtained from both stifles and submitted for cytological analysis (Table 2).

**Figure 3:**
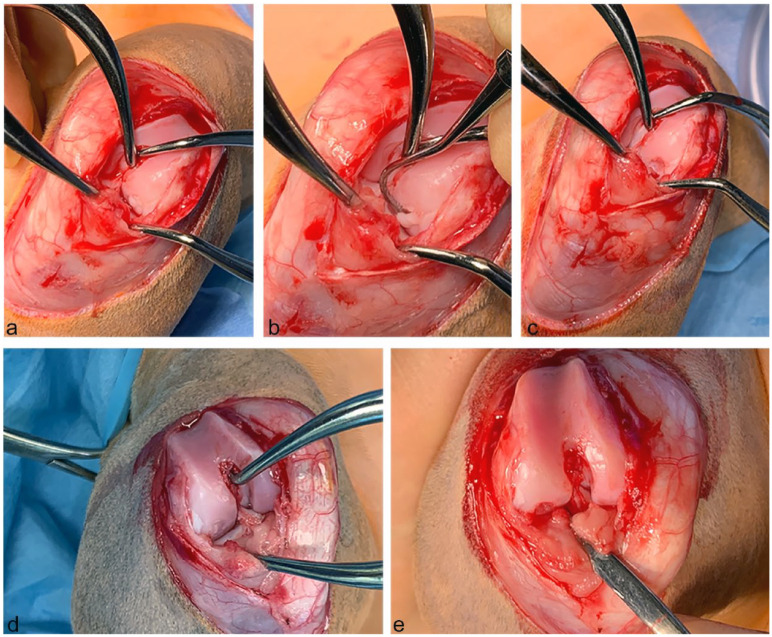
Intraoperative views of case 1, a male castrated Maine Coon cat at 15 months of age: (a) left stifle showing the osteochondral flap in situ; (b) the osteochondral flap elevated with a dental pick in the left stifle; (c) the appearance of the subchondral defect after flap removal but before curettage in the left stifle; (d) right stifle showing the osteochondral flap; and (e) the appearance of the subchondral defect after flap removal and curettage in the left stifle

Owing to the persistent lameness despite rest and NSAIDs, the decision was made to operate on the cat, and it was subsequently anaesthetised for bilateral stifle arthrotomies. A lateral parapatellar surgical approach was performed on the right stifle. An OCD lesion was located on the distal aspect of the lateral aspect of the femoral condyle (Figure 3). The flap was elevated using a dental pick and the remaining attachments were sharply resected. The OCD flap was placed in formalin and the underlying subchondral bone curetted. The stifle was flushed with sterile saline before closure. The joint capsule and fascia lata were closed in individual layers with an overlapping modified Mayo mattress suture pattern using 3-0 polydioxanone. This corrected the patellar laxity. Subcutaneous tissues and skin were closed using 4-0 poliglecaprone and simple interrupted skin sutures placed using 4-0 nylon.

The same surgical approach as described above for the right stifle was used for the left stifle. Upon manipulation, the left patella was only intermittently luxating and returned into the sulcus once released from being medially luxated. The OCD lesion on the left femoral lateral condyle had a more definite cartilaginous ‘flap’ appearance than the one on the right. It was also removed by elevation and sharp dissection of the remaining attachments followed by curettage of the underlying subchondral bed. The left OCD flap was placed in an individual formalin pot. Closure of the left stifle was similar to the right. After surgery, it was not possible to luxate either patella medially.

The cat was given methadone q6h for the first 24 h postoperatively and then analgesic cover was continued by injectable buprenorphine administered q6h for a further 24 h before the cat was discharged home on a 10-day course of meloxicam. The cat was weightbearing on both pelvic limbs on the day after surgery and it was discharged home with recommendations for strict cage rest for 3 weeks. After this, the cat was allowed access to the rest of the house.

The OCD flaps from both stifles were submitted for histological analysis and the results were highly suggestive of OC with areas of mineralisation and disruption of endochondral mineralisation (Table 2).

## Discussion

Stifle OC in cats affects young cats with a median age of 7 months and it can be bilateral or unilateral. The condition is seen more commonly in male cats; however, female cats can also be affected. It can affect a variety of breeds including DSH, Maine Coon and other purebred cats. Medial patellar luxation was a concurrent finding in several affected cats.

OC in dogs is most commonly reported in the shoulder and elbow.^15^ There are reports of humeral OCD in three male cats, a DSH, Burmese and Maine Coon.^7–9^ Resolution of the lameness occurred after surgical debridement in all cats.

In dogs, stifle OC is most commonly reported to affect the medial aspect of the lateral femoral condyle.^16^ In the cats reported herein, seven affected cats had lateral condylar lesions with three cats having unilateral medial condylar lesions.

Bilateral occurrence of stifle OC is common in dogs, reportedly affecting 63–72% of cases.^18,19^ This was less common in the cats reported herein, with only three (33%) bilaterally affected cats, although there were radiographic changes in the contralateral stifle in two of the cases reported as having unilateral OCD. In dogs, lameness is often unilateral even in cases with bilateral OCD.^16^

In our study, there were more male cats affected than female cats. In dogs, a similar increase in stifle OC in the male population has been reported.^16^ In another study, there was no reported difference between the incidence in male and female dogs.^20^

In dogs with stifle OC, the mean age at the onset of lameness has been reported as 5.9 months (range 3 months–3 years),^16^ although in a more recent canine study the median age at first diagnosis of OCD of the stifle joint was 2.62 years.^20^

Attribution of the early clinical signs of OC to other causes of hindlimb lameness in young dogs has been suggested as the reason for the late diagnosis.^19,21^ In several of the cats reported herein, the diagnosis occurred several months after the onset of clinical signs, likely because of the low incidence of OCD in the cat and therefore the low suspicion of its existence.

Dog breeds reported as affected by stifle OCD include the Irish Wolfhound, Labrador, Staffordshire Bull Terrier, German Shepherd Dog and Great Dane, with the majority being medium to large breeds.^15,16,20^ In our study, there was a variety of cat breeds affected, with two Maine Coon and five DSH cats. The affected purebred cats, the Maine Coon, Bengal, Devon Rex and Scottish Fold, are all large or relatively large breeds.

A subchondral deficit was visible on all radiographs of affected cats. In dogs, the lesion is reported to be clearer on the craniocaudal view and can be difficult to see on mediolateral views owing to superimposition of the condyles. In one of our reported cases, the lesions were initially missed on the mediolateral views.^18^ The fossa of the long digital extensor tendon, which is visible on the lateral femoral condyle, should not be confused with an OCD lesion.^18^ In the one cat described in detail in this case report, CT was instrumental in confirming a suspected diagnosis of stifle OCD with the suspicious flattening of the femoral condyles seen on the radiographs. In the canine shoulder, CT is readily able to identify osteochondral lesions that may have been missed on conventional radiography^22^ and CT used in the case of a young dog revealed femoral subchondral lesions that were not evident on radiography.^23^

Patellar luxation was a common concurrent condition affecting four of the cats with stifle OC reported herein. Patellar laxity is common in cats and it may be an incidental finding and not necessarily the cause of lameness. In a study looking at the association between medial patellar luxation and hip dysplasia in Maine Coon cats, medial patellar luxation was found in 45/78 (58%) cats and a conclusion was that clinically normal cats may have a certain degree of medial patellar subluxation.^24^ In the cats reported herein, only one had an osteotomy to specifically address the patellar luxation subsequent to a failure to improve after OCD fragment removal. In the two bilaterally affected cats, soft tissue imbrication or anti-rotational sutures were applied and the lameness improved in both cats although patellar luxation persisted in one case when the stifle was flexed. Of 116 dogs with stifle joint OC in one study, 76 (65.5%) had other stifle joint diagnoses; pain and other unspecific signs were the most common (n = 35, 30.2%) while cruciate ligament rupture was the most common subsequent diagnosis (n = 16, 13.8%). Patellar luxation was only recognised in one dog subsequent to the diagnosis of OC.^21^ No cats were recognised with cruciate ligament disease in this series of cases.

In dogs, the recommended treatment for OC varies depending on factors such as the affected joint, the severity of the clinical signs and the owner’s financial resources, and can be either conservative or surgical.^25^ Surgical treatment of OCD has traditionally involved removing the cartilage flap and stimulating defect healing by curetting the cartilage defect.^25^ The majority of cats in this series were treated by flap debridement and curettage of the underlying bone with successful outcomes reported as resolution of lameness or improvement in gait in all the cats with follow-up. The use of osteochondral grafts or synthetic grafts has been reported in dogs with stifle OCD^26,27^ but there are currently no reports of their use in cats.

The diagnosis of OC is based on signalment (age, breed and sex), history and physical and radiographic evidence.^28^ Other differential diagnoses for an osteochondral lesion include a non-displaced fracture, traumatic injury or a cruciate ligament avulsion.^11,23^ Potentially, the unilateral cases in the cats reported herein might be more likely to have been associated with a traumatic incident, particularly when a high grade (III) medial patellar luxation was present. However, in the three bilateral cases, and potentially the cases with unilateral lameness but bilateral radiographic changes, purporting a traumatic cause would be much harder to explain.

This is a retrospective study that includes some cases with incomplete follow-up data. However, given the rare nature of this condition, the collation of these partial data and addition to the sparse literature that has already been published was thought to be useful for disseminating information and raising awareness about this condition in cats.

## Conclusions

Stifle OC can be a cause of lameness in immature cats. It can occur concurrently with medial patellar luxation. Craniocaudal radiographic views or a CT scan of the stifle should be performed to investigate for its occurrence. Treatment by flap removal and curettage seems to achieve good results and recovery from lameness in the majority of cases. Conservative management in a smaller number of cases also resulted in a good outcome but some occasional stiffness.
